# Cost-Effectiveness of Physical Therapist Treatment in Addition to Usual Podiatry Management of Plantar Heel Pain: Economic Evaluation of a Randomized Clinical Trial

**DOI:** 10.1093/ptj/pzaf119

**Published:** 2025-10-03

**Authors:** Shane M McClinton, Bryan C Heiderscheit, Timothy W Flynn, Daniel Pinto

**Affiliations:** Doctor of Physical Therapy Program, Des Moines University, West Des Moines, IA 50266, United States; Departments of Orthopedics & Rehabilitation and Biomedical Engineering, University of Wisconsin-Madison, Madison, WI, United States; Own My Health, Larkspur, CO, United States; Department of Physical Therapy, College of Health Sciences, Marquette University, Milwaukee, WI, United States

**Keywords:** Cost-effectiveness, Physical Therapy, Plantar Fasciitis, Podiatry, Quality of Life

## Abstract

**Importance:**

Plantar heel pain (PHP) contributes to reduced quality of life and is costly to manage. Persons with PHP are infrequently referred to a physical therapist after presenting to primary care or podiatry.

**Objective:**

The study objective was to compare the cost-effectiveness of usual podiatry care (uPOD) plus physical therapist treatment with that of uPOD alone in the management of PHP.

**Design:**

A cost-effectiveness analysis from societal and health care sector perspectives and a 3-year time horizon was performed alongside a randomized clinical trial. Intention to treat was used as the base case, and sensitivity analyses were used to assess the impact of adherence to treatment (ie, per protocol) and PHP-specific costs.

**Setting:**

The setting was a multidisciplinary outpatient clinic in the United States.

**Participants:**

Participants were 95 eligible patients with PHP.

**Interventions:**

uPOD consisted of a stretching handout, medication, injections, and orthotics; uPOD plus physical therapist treatment also included physical therapist intervention consisting of manual therapy, exercise, foot taping, and iontophoresis.

**Main Outcomes and Measures:**

Cost-effectiveness was determined by between-group differences in costs relative to quality-adjusted life-years (QALYs). Cost-effectiveness at different thresholds of decision maker willingness to pay was illustrated using the cost-effectiveness acceptability curve.

**Results:**

uPOD plus physical therapist treatment reduced societal costs by $2708 (95% CI = −$294 to $5709) relative to uPOD and increased QALYs by 0.09 (95% CI = −0.01 to 0.18). The cost-effectiveness acceptability curve demonstrated 98%, 99%, and 97% probabilities of cost-effectiveness of uPOD plus physical therapist treatment in the base-case, per-protocol, and PHP-specific cost analyses using a willingness-to-pay threshold of $50,000 per QALY.

**Conclusions:**

Adding physical therapist treatment to uPOD lowered total costs and improved quality of life despite increased short-term health care utilization. Results were not altered when considering adherence to treatment or PHP-specific costs.

**Relevance:**

This study informs shared decision-making between providers and patients with PHP about the costs and benefits of adding physical therapist treatment and provides support for the economic value of physical therapist treatment for PHP.

## INTRODUCTION

Plantar heel pain (PHP) affects 0.5% to 10% of the general population^1–5^ and 5% to 18% of athletes,^6^^,^^7^ with an annual health care cost of PHP in the United States estimated at $285 to $558 million (inflated from 2007 to 2023 United States dollars).^8^ Persons with PHP has pain-related interference with weight-bearing activities, including work, walking, and running,^5^^,^^9^ that contribute to reduced quality of life, disability, and falls.^9–11^ PHP is associated with debilitating conditions including back pain, joint problems, neuropathic pain, depression, diabetes, headaches, and sleep disorders.^5^^,^^12^^,^^13^ Although 54% to 82% of persons with PHP will recover, 18% to 46% have persistent symptoms and limited function and require ongoing treatment.^14–16^

Persons with PHP access the health care system via different providers. Many patients will seek assistance from, or be referred to, a podiatrist to manage their PHP. Because persons with PHP often have persistent symptoms and concurrent movement-related impairments affecting their health and contributing to increased long-term management costs, interdisciplinary collaboration is often warranted. Although there is regional variation and overlap in practice scope, Podiatrists commonly emphasize foot orthoses, shoe advice, heel pads, medication, and injections to manage heel pain.^17^ When podiatrists collaborate with physical therapists, the physical therapist may complement podiatric care with more specific exercise instruction, manual therapy and treatment of local and proximal impairments (eg, low back pain).^17^^,^^18^ Although multidisciplinary care models are promoted in health care, and physical therapists specialize in management of PHP-related impairments, physical therapists are not commonly utilized early in the management of PHP. Although the referral rate from podiatrists is unknown, the referral rate from physicians to a physical therapist for PHP is only 7% to 20%.^19^^,^^20^ The low referral rate may be due to concerns about additional short-term costs when utilizing physical therapists to assist with PHP treatment, although there is potential to reduce long-term costs using a multidisciplinary care model. Therefore, the research question was whether the addition of physical therapist treatment to usual podiatry care (uPOD) for PHP is cost-effective.

## METHODS

### Study Design and Participants

This was a cost-effectiveness analysis conducted alongside a randomized clinical trial that compared uPOD with uPOD plus physical therapist intervention. The protocol and clinical outcomes of this trial were reported elsewhere.^21^^,^^22^ Participants (18–70 years old) were recruited from the Des Moines University Foot and Ankle Clinic in Des Moines, Iowa, USA, between April 2014 and October 2016. Participants were included if they were diagnosed with PHP by the podiatrist. Participants were excluded if they had minimal functional limitations (score on the activities of daily living subscale of the Foot and Ankle Ability Measure of >88/100); symptoms for longer than 1 year; surgery of the foot, ankle, or lower leg; clinical signs of radiculopathy; contraindications to manual therapy interventions; clinical indication of plantar fascia rupture; treatment for PHP in the preceding 6 weeks; or an inability or unwillingness to complete questionnaires or recommended treatment. The cost-effectiveness analysis included societal and health care sector perspectives. The societal perspective included costs to the health care system (health care visits and medication), patient, family or friends (transportation to/from appointments, copayment/coinsurance, housework/yardwork assistance, foot orthoses, devices to help symptoms), and productivity (absenteeism and presenteeism). The health care sector perspective included costs related to health care visits, medication, and copayment/coinsurance.^23^ A 3-year time horizon was used to capture the treatment costs and consequences of PHP including recalcitrant patients as 18% to 46% of patients with PHP have persisting symptoms an average of 2 years after treatment.^14–16^

### Ethics Approval

Institutional review board approval was received from the Des Moines University (IRB ID 04–13-02) and Rocky Mountain University of Health Professions (protocol number 131034–02) review boards. All participants provided written informed consent to participate and written informed authorization to use and disclose individual health information for research purposes before data collection began.

### Interventions

Participants were randomized to uPOD or uPOD plus physical therapist treatment using a concealed and blocked allocation method.^21^^,^^22^ In both groups, the patient was first seen by a podiatrist who evaluated the patient and provided treatment at his or her discretion; treatment may have included anti-inflammatory medication or injection, instruction on specific exercises, orthotic prescription, or education on proper footwear.^21^ Individuals in the uPOD group continued to follow-up with the podiatrist. Individuals assigned to receive uPOD plus physical therapist treatment received further evaluation and treatment from a physical therapist and followed up with the podiatrist only if needed. Treatment for the individuals receiving uPOD plus physical therapist treatment consisted of manual therapy (talocrural, rear foot, calf, and plantar foot mobilization/manipulation), specific calf and foot stretching and strengthening exercises, foot taping, correction of contributing factors (such as proximal neurological or musculoskeletal impairments), or iontophoresis.^21^^,^^24^ Physical therapists were guided by a manual of procedures that included a 3-phase progression (symptom modulation, graded exercise, and return to function) that was impairment based, informed by short-term treatment response, and centered on individual patient goals. Interventions were prioritized on the basis of the most relevant local and proximal impairments and informed by PHP practice guidelines and current best evidence (Suppl. Figure 1). Previously published work provides a detailed account of the intervention and clinical decision-making of the physical therapist treatment used in this clinical trial.^24^

### Health Outcomes

The primary health outcome used to calculate cost-effectiveness was quality-adjusted life-years (QALYs). The EuroQol 5-dimension 3-level questionnaire (EQ-5D-3L) was used to capture health utility converted to a single index anchored from 0 (death) to 1 (perfect health) using a US preference–weighted value set.^25^^,^^26^ The EQ-5D-3L is a standardized measure of health status that uses responses from a questionnaire to rank 5 health dimensions (mobility, self-care, usual activities, pain/discomfort, and anxiety/depression) to 3 health levels. The EQ-5D-3L was completed at baseline, 6 weeks, 6 months, 1 year, 2 years, and 3 years after the initiation of treatment. Patient-level QALYs were estimated at 1, 2, and 3 years using time-weighted averages of the EQ-5D-3L index score measured at the beginning and end of each measurement period.^27^ For example, QALYs for the first year were calculated as follows (where BL = baseline and EQ5D = EQ-5D-3L):


\begin{align*} QALY=&\ \Bigg[\frac{\left( EQ5{D}_{BL}+ EQ5{D}_{6 wk}\right)}{2}\frac{1.38}{12}\\&+\frac{\left( EQ5{D}_{6 wk}+ EQ5{D}_{6 mo}\right)}{2}\frac{4.62}{12}\\&+\frac{\left( EQ5{D}_{6 mo}+ EQ5{D}_{1 yr}\right)}{2}\frac{6}{12}\Bigg]. \end{align*}

QALYs for years 2 and 3 were calculated similarly and represent the sum of the current and prior year’s QALYs. QALYs for years 2 and 3 were not discounted to avoid double discounting and underestimation of health outcomes because the US preference–weighted value set was derived using the time trade-off valuation technique, which accounts for time preference.^26^^,^^28^ Additionally, the numeric pain rating scale and the activities of daily living subscale of the Foot and Ankle Ability Measure were measured at baseline and each follow-up.^21^^,^^22^

### Resource Utilization and Costs

Costs are reported in 2023 US dollars and included health care system costs (health care utilization including provider visits and medication), disease-related costs (copayment/coinsurance, transportation, housework/yardwork assistance, and devices), and productivity costs (absenteeism and presenteeism). Additionally, costs specific to PHP treatment were separated for sensitivity analysis. PHP-specific costs were defined as costs allocated to PHP International Classification of Diseases, Tenth Revision, billing codes (M72.2 or M479.67 [if confirmed by chart review]) or per-patient report that a service or device was primarily for the patient’s PHP. Health care utilization and costs were obtained directly from billing records for all visits at the Des Moines University Foot and Ankle Clinic. Health care utilization outside of this clinic in addition to disease-related and productivity costs/consequences was captured using a questionnaire (the Plantar Heel Pain Cost and Consequences Questionnaire [PCQ]) (Suppl. Material) modified after the Osteoarthritis Cost and Consequences Questionnaire^29^ and administered at baseline, 6 weeks, 6 months, 1 year, 2 years, and 3 years after the initiation of treatment. The PCQ included questions about absence from paid work (absenteeism) and reduced productivity at paid work (presenteeism) and was based on the Health and Labour Questionnaire.^30^ In addition, participants reported their hourly wage to value productivity using a human capital approach.^31^ Costs for utilization captured from the PCQ were derived from provider-specific average costs per visit from data obtained from Des Moines University Foot and Ankle Clinic billing records where applicable and secondarily from complementary and alternative medicine use among US adults from the National Health Interview Survey.^32^ The Medicare Fee Schedule,^33^ Internal Revenue Service standard mileage rate,^34^ and US Bureau of Labor Statistics^35^ were used to estimate procedures (eg, surgery), transportation, productivity, and housework/yardwork help costs, respectively. Medication costs were estimated using medication-specific data from the National Average Drug Acquisition Cost.^36^ To account for inflation, costs were converted to a common time (2023) using gross domestic product price deflators.^37^ To account for time preference, costs beyond the 1-year follow-up were discounted at the rate of 3% per year as recommended by the Second Panel on Cost-Effectiveness in Health and Medicine.^23^

### Data Analysis

Baseline group variables were summarized using the mean and SD for continuous measures and percentages for categorical measures. An intention-to-treat analysis was used as the base case. Unless otherwise noted, data analysis was performed using Stata v17.0 (StataCorp LLC, College Station, TX, USA).

#### Missing Data

Missing data were imputed using k-nearest neighbors.^38^^,^^39^ The R (R Foundation for Statistical Computing, Vienna, Austria) packages naniar and visdat were used to assess randomness of missing data, and the VIM package of R was used to impute missing data using k-nearest neighbors.

#### Cost-Effectiveness Analyses

Separate multivariable analyses were performed to estimate total cumulative costs and QALYs for the 3-year study period using generalized linear models including baseline values (costs or EQ-5D-3L), age, sex, weight, symptom duration, and household income. Link functions and families for the generalized linear models were selected on the basis of the fit of the data using custom Stata programs integrating the modified Park, Pearson correlation, Pregibon, and modified Hosmer and Lemeshow tests.^40–42^ Costs for subcategories of total cost and secondary outcomes were summarized using the mean and bootstrapping (3000 replications) to report the SE.

#### Sampling Uncertainty

SEs and 95% CIs were estimated by a nonparametric bootstrap (3000 replications) from univariate (cost subcategories) and multivariate (total cost and QALY) analyses. The results of the nonparametric bootstrap were used to calculate incremental cost-effectiveness ratios [ICER, ie, (costs of uPOD plus physical therapist treatment − costs of uPOD)/(QALY of uPOD plus physical therapist treatment − QALY of uPOD)], to illustrate uncertainty around the ICER using the cost-effectiveness plane, and to illustrate cost-effectiveness at different thresholds of decision maker willingness to pay using the cost-effectiveness acceptability curve. Treatment was deemed cost-effective if the ICER was less than a willingness-to-pay (WTP) threshold of $50,000 per QALY.^43–45^

#### Sensitivity Analysis

A 1-way sensitivity analysis was performed to assess the impact of adherence to treatment (ie, per-protocol analysis) and PHP-specific costs on cost-effectiveness estimates relative to the base-case analysis (intention to treat and all costs). The per-protocol analysis excluded individuals who did not meet the criteria for the completion of treatment, including attendance at all clinic appointments/follow-up assessments according to the plan mutually set by the patient and provider and, for individuals receiving uPOD plus physical therapist treatment, attendance at 4 or more visits if warranted.^21^ The PHP-specific costs included the same data set as the base case but excluded all costs not primarily associated with the management of PHP.

### Role of the Funding Source

The funders played no role in the design, conduct, or reporting of this study.

## RESULTS

### Participants

Participant characteristics for the base-case (which included the same participants for the PHP-specific cost analysis) and per-protocol analyses are summarized in Table 1. The participant flow diagram includes the number of participants that completed follow-up and were analyzed at each time point (Suppl. Figure 2). There were no missing baseline data or data missing related to resource use and costs associated with services provided at the study location. For patient-reported resource use that was used to calculate cost outcomes and health outcomes (ie, those reported in Table 2) at 1, 2, and 3 years, 4.5%, 13.3%, and 11.6% of all cost-related outcome data, respectively, and 7.4%, 21.1%, and 18.9% of outcome-related data, respectively, were missing. The maximum percentages missing from any single variable were 30.5% for cost outcomes and 21.1% for health outcomes, both observed in year 2 reporting. The total (all 3 years combined) percentage of data missing and imputed for use in multivariate analysis was 8.6%.

**Table 1:** Characteristics of All Participants and Participants Who Completed Treatment (per Protocol) in Each Group*^a^*

**Characteristic**	**Base Case**		**Per Protocol**	
	**uPOD + Physical Therapy (n = 48)**	**uPOD (n = 47)**	**uPOD + Physical Therapy (n = 38)**	**uPOD (n = 41)**
Age, y	49.8 (10.8)	50.3 (10.3)	51.1 (10.7)	50.9 (10.1)
Females, no. (%) of participants	38 (79.2)	33 (70.2)	30 (78.9)	29 (70.7)
Height, cm	170.1 (8.8)	170.6 (8.7)	169.6 (9.4)	170.5 (8.4)
Weight, kg	92.6 (24.1)	91.1 (21.5)	88.9 (18.8)	92.6 (21.7)
BMI, kg/m^2^	32 (7.6)	31.3 (6.9)	31 (6.8)	31.8 (7.1)
Duration of symptoms, d	129.2 (105.9)	147.2 (111.1)	126.4 (110.9)	137.3 (97.7)
Household income, US $	93,467.27 (63,615.55)	104,281.70 (69,638.60)	89,463.92 (62,071.44)	100,142.44 (67,880.43)
Education level, no. (%) of participants				
High school	4 (8.3)	2 (4.3)	2 (5.3)	2 (4.9)
Some college/no degree	8 (16.7)	8 (17)	6 (15.8)	7 (17.1)
Associate’s degree	5 (10.4)	7 (14.9)	4 (10.5)	6 (14.6)
Bachelor’s degree	15 (31.3)	20 (42.6)	12 (31.6)	18 (43.9)
Master’s degree	14 (29.2)	7 (14.9)	13 (34.2)	6 (14.6)
Doctorate	2 (4.2)	3 (6.4)	1 (2.6)	2 (4.9)
Occupation*^b^*, no. (%) of participants				
Health care/personal care/fitness*^c^*	6 (12.5)	2 (4.3)	4 (10.5)	2 (4.9)
Management/business/computer/legal*^d^*	14 (29.2)	18 (38.3)	11 (28.9)	16 (39)
Education/library/ministry*^e^*	9 (18.8)	7 (14.9)	8 (21.1)	6 (14.6)
Clerical/sales/service*^f^*	9 (18.8)	10 (21.3)	6 (15.8)	8 (19.5)
Construction/maintenance/material moving*^g^*	1 (2.1)	3 (6.4)	0	3 (7.3)
Not in paid work	9 (18.8)	7 (14.9)	9 (18.8)	6 (14.6)

^a^ Values are reported as mean (SD) unless otherwise indicated. Abbreviations: BMI = body mass index; uPOD = usual podiatry care.

^b^ According to major job categories defined by the US Bureau of Labor Statistics.^35^

^c^ Includes health care practitioners and technical, health care support, and personal care and service occupations.

^d^ Includes management, business and financial operations, computer, and legal occupations.

^e^ Includes community and social services, educational instruction and library, and arts, design, entertainment, sports, and media occupations.

^f^ Includes protective services, food preparation and serving occupations, sales occupations, and office and administrative support occupations.

^g^ Includes construction, installation, maintenance and repair, and transportation and material-moving occupations.

**Table 2:** Total Costs and Effects and Incremental Cost and Effect Differences for uPOD Plus Physical Therapist Treatment and uPOD Alone*^a^*

**Perspective, Analysis Case, and Group**	**Cost, US $, Mean (SE)** ^***b***^	**Incremental Cost Difference (95% CI)**	**Effectiveness, QALY, Mean (SE)**	**Incremental Effectiveness (95% CI)**	**ICER**
Societal perspective					
Base case					
uPOD + physical therapy	4650.06 (808.18)	−2707.68 (−5709.45 to 294.09)	2.69 (0.04)	0.09 (−0.01 to 0.18)	Dominant*^c^*
uPOD	7357.73 (1344.01)		2.61 (0.03)		
Per protocol					
uPOD + physical therapy	5354.59 (1093.41)	−1779.13 (−4843.23 to 1284.99)	2.72 (0.05)	0.12 (−0.01 to 0.23)	Dominant*^c^*
uPOD	7134.47 (1207.09)		2.6 (0.04)		
PHP-specific costs					
uPOD + physical therapy	1522.06 (268.15)	−574.54 (−1601.66 to 452.59)	2.69 (0.04)	0.09 (−0.01 to 0.18)	Dominant*^c^*
uPOD	2096.66 (451.27)		2.61 (0.03)		
Health care sector perspective					
Base case					
uPOD + physical therapy	4453.44 (853.70)	−1140.27 (−3506.76 to 1226.22)	2.69 (0.04)	0.09 (−0.01 to 0.18)	Dominant*^c^*
uPOD	5593.71 (1011.82)		2.61 (0.03)		
Per protocol					
uPOD + physical therapy	4799.34 (1089.32)	−1121.76 (−4090.02 to 1846.49)	2.72 (0.05)	0.12 (−0.01 to 0.23)	Dominant*^c^*
uPOD	5921.11 (1146.35)		2.6 (0.04)		
PHP-specific costs					
uPOD + physical therapy	1125.76 (176.91)	288.39 (−9.16 to 585.94)	2.69 (0.04)	0.09 (−0.01 to 0.18)	2490.30 (2396.00 to 2584.61)
uPOD	837.37 (121.49)		2.61 (0.03)		

^a^ Abbreviations: ICER = incremental cost-effectiveness ratio; PHP = plantar heel pain; QALY = quality-adjusted life-year; uPOD = usual podiatry care.

^b^ Cumulative costs over a 3-year time horizon where years 2 and 3 costs were discounted 3% and inflated from 2016 to 2023 US dollars.

^c^ Treatment dominated usual care (ie, uPOD + physical therapy cost less and was more effective than uPOD).

### Resource Utilization

uPOD plus physical therapist treatment showed a trend towards utilizing more physical therapist visits in year 1 (eg, in the base case, 4.1 visits for uPOD plus physical therapist treatment vs 1.87 for uPOD alone) and more health care visits than uPOD in 7 of the 9 health care service categories throughout the study (ie, more visits [mean difference in visits between uPOD plus physical therapist treatment and uPOD] with a physical therapist [2], family practice physician [0.04], osteopathic physicians [0.08], acupuncturist [0.14], chiropractor [2.23], massage therapist [0.89], or reflexologist [0.04] but fewer visits with an orthopedic physician [0.12] or podiatrist [0.45]) (Suppl. Table 1). Conversely, uPOD utilized more medication (mean difference in total number of pills = 76.82) and more housework/yardwork help (mean difference = 2.29 hours) throughout the study than uPOD plus physical therapist treatment. In addition, uPOD reported more hours in productivity loss (mean difference in absenteeism and presenteeism = 3.71 hours) than uPOD plus physical therapist treatment.

### Cost-Effectiveness

**Figure 1:**
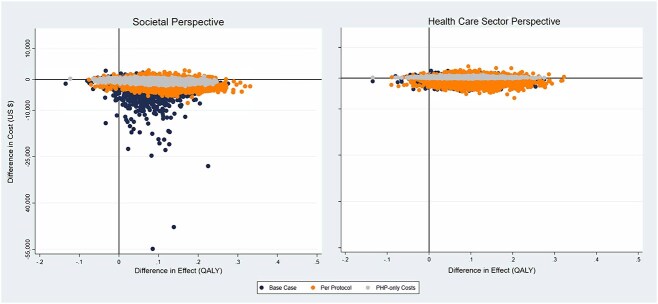
Cost-effectiveness plane of usual podiatry care (uPOD) plus physical therapist treatment relative to uPOD alone from societal and health care perspectives in United States dollars (US $) per quality-adjusted life-year (QALY) gained for the base-case and the per-protocol and plantar heel pain (PHP)–only cost sensitivity analyses. For the base-case, per-protocol, and PHP-specific cost analyses, uPOD plus physical therapist treatment was dominant (lower costs and more effective), with 92%, 85%, and 85% of replicates in the southeast quadrant of the cost-effectiveness plane.

**Figure 2:**
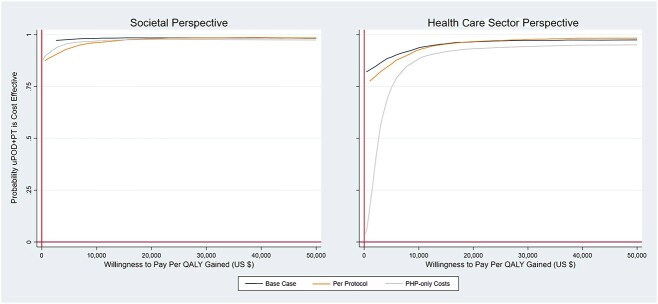
Cost-effectiveness acceptability curve of usual podiatry care (uPOD) plus physical therapist treatment (uPOD+PT) relative to uPOD from societal and health care sector perspectives in United States dollars (US $) per quality-adjusted life-year (QALY) gained for the base-case and the per-protocol and plantar heel pain (PHP)–only cost sensitivity analyses.

From a societal perspective and a 3-year time horizon, uPOD plus physical therapist treatment had lower costs and was more effective in improving quality of life than uPOD in the base-case and sensitivity analyses, as demonstrated by incremental cost and effect differences (Table 2) and the number of bootstrap replicates landing in the lower right quadrant of the cost-effectiveness plane (Figure 1). Furthermore, for the base-case, per-protocol, and PHP-specific cost analyses, uPOD plus physical therapist treatment was dominant (lower costs and more effective), with 92%, 85%, and 85% of replicates in the lower right or southeast quadrant of the cost-effectiveness plane, respectively; had lower costs, with 96%, 87%, and 87% of replicates below the line of no cost difference, respectively; and was more effective, with 96%, 98%, and 96% of replicates to the right of the line of no effect difference, respectively. The probabilities that uPOD plus physical therapist treatment was cost-effective at the WTP threshold of $50,000 per QALY gained for the base-case, per-protocol, and PHP-specific cost analyses were 98%, 99%, and 97%, respectively (Figure 2). The cost-effectiveness acceptability curve also illustrates a high probability of uPOD plus physical therapist treatment being cost-effective at low WTP thresholds. For example, 95% confidence of the effectiveness of uPOD plus physical therapist treatment was achieved at a WTP threshold of $2589 per QALY in the base case ($6850 in the per-protocol and $4682 in the PHP-specific cost analyses).

Slightly higher incremental effectiveness was observed in the per-protocol analysis than in the base-case analysis (Table 2). When exploring the data, individuals who did not complete uPOD plus physical therapist treatment had slightly lower total costs ($3736) than individuals who completed treatment (ie, per protocol, $4302) but also had lower QALYs (non-completers = 2.61 vs per protocol = 2.72). In contrast, individuals not completing uPOD had lower total costs ($1861) than individuals who completed treatment ($5610) but achieved similar QALYs (non-completers = 2.66 vs per protocol = 2.6). A significant contribution to the total costs for individuals receiving uPOD plus physical therapist treatment were from 2 participants; both did not complete treatment. One participant was randomized to uPOD plus physical therapist treatment, never attended a physical therapist appointment, and went on to be 1 of 2 participants with significant costs due to PHP-related surgery. The second participant attended only 2 physical therapist appointments and had significant costs related to bariatric surgery.

**Figure 3:**
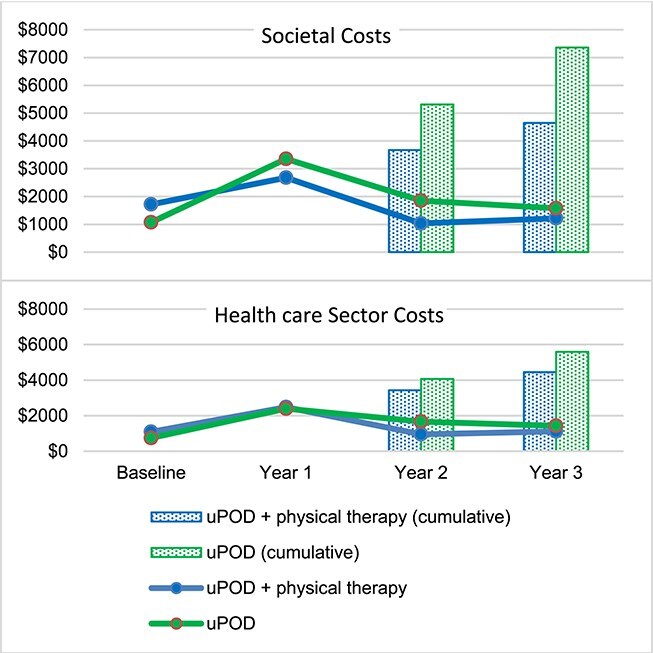
Total societal (top) and health care sector (bottom) costs per year (solid lines) and cumulative 2- and 3-year total costs (dotted bars) for usual podiatry care (uPOD) plus physical therapist treatment (uPOD+PT) and uPOD alone in the base case.

**Figure 4:**
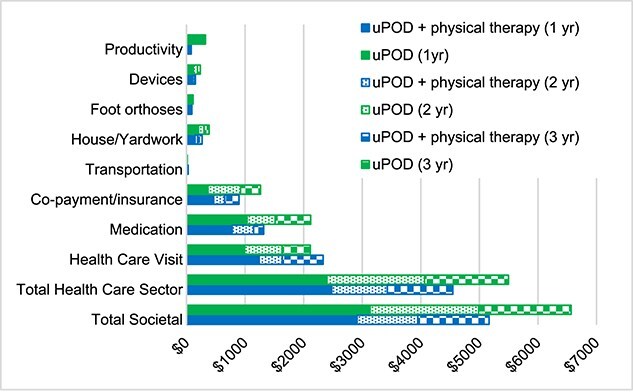
Costs for each category (including total costs from societal and health care sector perspectives) per year for usual podiatry care (uPOD) plus physical therapist treatment (uPOD+PT) and uPOD alone in the base-case analysis.

The majority of PHP-specific costs and non–PHP-specific costs were incurred in year 1 and were lower in years 2 and 3 (Figure 3; Suppl. Table 2). Despite increased physical therapist utilization and health care visit costs in year 1, uPOD plus physical therapist treatment demonstrated cost-effectiveness relative to uPOD at year 1 (Figure 3; Suppl. Table 2). The largest contribution to year 1 total costs were health care utilization (uPOD plus physical therapist treatment = 47.4%; uPOD = 50.2%), including 23.1% and 19.1% of total costs for PHP treatment for uPOD plus physical therapist treatment and uPOD alone, respectively. In years 2 and 3, <3.5% of costs for health care utilization were PHP related and persons in the uPOD group had higher costs related to medication utilization, copayment/coinsurance, housework/yardwork help, devices to help symptoms/function, and productivity loss (Figure 4**;**  Suppl. Table 2).

When cost-effectiveness was analyzed from a health care sector perspective, results mirrored the societal perspective (ie, uPOD plus physical therapist treatment had lower costs and was more effective in improving quality of life than uPOD) (Table 2; Figures 1 and 2), with the exception of the PHP-specific sensitivity analysis. In the PHP-specific analysis from a health care sector perspective, uPOD plus physical therapist treatment demonstrated more PHP-related costs in year 1 (Suppl. Table 2), resulting in greater cumulative costs over the 3-year horizon; however, the greater effectiveness of uPOD plus physical therapist treatment resulted in an ICER of $2490 (95% CI = 2396 to 3585) per QALY gained—well below the $50,000 per QALY WTP threshold.

## DISCUSSION

The results of this study indicate that uPOD plus physical therapist treatment was cost-effective despite an increase in total and PHP-specific health care visit costs that was associated with increased health care services utilization, mostly in year 1. Despite more health care visit costs, uPOD plus physical therapist treatment demonstrated lower costs related to medication, copayment/coinsurance, housework/yardwork help, foot orthoses, devices to manage symptoms, and productivity loss. After the first year, costs for conditions other than PHP were greater in the uPOD group that was associated with increased medication utilization and greater copayment/coinsurance costs. For example, in the base case, medication costs for conditions other than PHP increased by an average of $136 per participant between years 2 and 3 in the uPOD group, whereas costs decreased by $193 in individuals receiving uPOD plus physical therapist treatment. This is consistent with others that reported 41% of persons with PHP used medication for pain, but only 6% used pain medication specifically for PHP.^5^ The interaction between PHP and comorbid conditions appears to contribute to health care utilization and costs. In this study, long-term costs (ie, >1 year) for management of conditions not directly billed as for PHP was lower with the addition of physical therapist treatment.

It is common for persons with PHP to have a comorbid painful neurological or musculoskeletal condition^5^^,^^12^^,^^46^^,^^47^ and to have difficulty staying active via running or walking.^9^^,^^48^ Individuals with PHP often have excess body mass and are more likely to have diabetes, depression, and sleep disorders.^5^^,^^49^^,^^50^ The longer a person has PHP, chances of PHP severity increases and response to physical therapist treatment lessens.^5^^,^^51^ Therefore, delays in effective PHP management may affect resource utilization, the ability to stay active, and the economic burden of concomitant disease management. This study demonstrates that more costs to manage PHP initially (ie, via addition of physical therapist treatment early after presentation to a podiatrist) may have downstream savings in the management of PHP and other comorbid conditions.

Participants allocated to uPOD plus physical therapist treatment received physical therapist intervention early (~14 days) after initial presentation to a podiatrist,^21^ but after having symptoms for 4.5 (SD = 3.5) months, on average. Individuals with symptoms for >1 year were excluded from participating on the basis of literature at the outset of this study indicating diminished outcomes to conservative treatment in persons with longer symptom duration.^14^^,^^52^ This exclusion criterion contributed to the mean duration of symptoms in this sample being shorter than the average of 16.2 months found in other PHP studies.^53^ The cost-effectiveness of uPOD plus physical therapist treatment in a more chronic population (eg, symptoms for more than 1 year) is unknown, but the physical therapist interventions of this pragmatic trial are applicable to chronic PHP presentations. Patients with chronic symptoms can benefit from uPOD plus physical therapist treatment, but patients with symptoms longer than 7 months were found to be 4 times less likely to have success with physical therapist treatment,^51^ highlighting the importance of early referral to a physical therapist. The results of early physical therapist intervention from this trial are consistent with other investigations that demonstrate decreased costs and reduced health care utilization (opioid medication, advanced imaging, surgery, injections and episode duration) when physical therapist intervention is provided early versus being delayed for musculoskeletal disorders.^54^^,^^55^

In this study, uPOD plus physical therapist treatment reduced costs associated with work and home roles in addition to the need to purchase symptom-relieving products. On the basis of prior work, 53% of individuals with PHP reported that pain interfered with normal work activities, with many individuals needing to use sick leave because of the condition.^5^^,^^48^ Physical therapist management added to initial presentation to a podiatrist may help reduce this burden from a personal and societal perspective. The results of this investigation may inform podiatrists, patients, and physical therapists in a shared decision-making context when considering the costs (short- and long-term) and benefits of adding physical therapist to podiatry treatment. For example, if providers or patients are reluctant to add physical therapist treatment to initial podiatry care, this study indicates the greater short-term direct and indirect costs incurred by the patient and those to the health care sector are recouped between 1 and 2 years and resulted in lower health care sector and societal costs and improved health outcomes over the longer term. Consistent results from a health sector and societal perspective provides further support to patients and payers that adding or authorizing physical therapist treatment after initial treatment from a podiatrist is a good allocation of resources to achieve improved health outcomes.

### Limitations

The results of this trial should be interpreted in light of its limitations. The repeatability and generalizability beyond the single clinical location of this study (ie, US-based multidisciplinary clinic that comprised multiple public and private third-party payers) that included a relatively small sample of persons with PHP is yet to be determined. Despite the single location, the pragmatic nature of this trial that was guided by clinical guidelines and current PHP evidence which enhances the generalizability under real world conditions.^56^ Although this study included podiatrists as the primary access point for PHP care, many persons with PHP are seen initially by a primary care physician,^5^^,^^13^^,^^19^ and it is not known if these findings would be replicated with physical therapist treatment provided after usual care from a primary care physician. In addition, many patients access care directly from physical therapists without initial care from a podiatrist and in a direct-access scenario, physical therapist treatment may include interventions provided by podiatrists in this study (eg, foot orthoses, shoe advice and, in some jurisdictions, medication and injections). This study reflects a health care environment where physical therapists do not typically prescribe medication or provide injections and where podiatrists do not routinely provide manual interventions, treatment of impairments proximal to the lower leg, and more than unsupervised exercise recommendations. Therefore, the results of this study are only applicable to circumstances when physical therapist treatment is added to care that is initially provided by a podiatrist where both practitioners treat in practice patterns that reflect the health care environment reported in this study. There is overlap in the scope and patterns of practice between physical therapists and podiatrist and therefore more pragmatic trials like this are needed to confirm cost-effectiveness of collaboration between these providers in other locations. Additionally, further research is warranted that considers the potential that physical therapist treatment may be more cost-effective when patients initiate care with a physical therapist who also prescribes medication and performs injections as it is likely to change incentive structures and multidisciplinary care patterns.

The findings of this study are limited to the cost categories captured by the study. Recommended cost categories were considered along with variables that had potential to affect the results on the basis of PHP research and input from experts in economic evaluation, public health, PHP management, and individuals with PHP.^57^^,^^58^ Although we attempted to capture all relevant costs, there may be variables not represented in this study. For example, there is emerging evidence of psychosocial factors associated with PHP and increased mental health services utilization by persons with musculoskeletal disorders.^48^^,^^59–62^ We did not specifically capture mental health utilization although psychologically-informed interventions may have been provided. In addition, costs related to loss of participation in recreational activities (eg, running, sports, etc.) were not estimated and self-reported utilization and costs obtained via the PCQ (Suppl. Material) are contingent on the accuracy and recall of the participant, particularly at the 2- and 3-year follow-up assessments. At the 2- and 3-year follow-up assessments, the PCQ asked participants to recall the previous year’s PHP-related costs for health care utilization outside of the study location, medication, PHP-specific device purchases, work productivity, and housework/yardwork assistance costs. A 1-year recall may underestimate total utilization and costs of the sample and typically affects less salient health service use but is very unlikely to miss higher-cost items, such as surgery, that would drive cost estimates. In addition, there is no evidence that we are aware of indicating the 2 groups would differ in recreational participation, mental health utilization, or recall bias that would substantially affect the conclusions.

In this study, we prospectively collected patient-level resource use and costs and used bootstrapping of the imputation and estimation to reduce artificial reduction in estimates of sampling uncertainty.^58^ Although the total missing data from all variables was 8.6%, 2 variables (year 2 absenteeism and presenteeism) were missing in 30% of cases. Because of the minimal cost contribution of these productivity variables to total costs after year 1 and similar missingness between groups, it is unlikely to have a significant impact on the results. In addition, a 1-way sensitivity analysis was performed to assess the robustness of the results to the influences of adherence to treatment and limiting costs to those more directly related to PHP. Future analysis may consider 1- or 2-way probabilistic analysis using a larger and more diverse sample (ie, from more than 1 clinical location) incorporating current and country-specific estimates, such as medication cost, fee schedules, and productivity loss.

The timing of this economic evaluation relative to the completion of the clinical trial is within the range of delays observed in reporting of economic evaluations. For clinical trials that planned to conduct an economic evaluation, the majority had not published results until after a mean of 6.5 years since the trial end and trials with cost-effectiveness publications were published a mean of 3 and a maximum of 7.9 years after the trial ended.^63^ Similarly, ~50% of economic evaluations conducted alongside clinical trials in Australia were not published within 5 years of trial completion and economic evaluations that were published in this time were published 2 (interquartile range = 2–3) years after the trial ended.^64^ This trial was hampered by the lack of federal funding including support for a health economist which has been shown to enhance economic evaluation publication.^64^ In addition COVID-19 circumstances, changes to the lead author’s professional obligations, and the peer review process affected publication timing.^65^ Challenges to timely publication of economic evaluations conducted alongside non–federally funded clinical trials underscores the need to build the capacity to conduct and support economic analysis including the peer-review processes.^64^^,^^65^ To address the timing of this evaluation relative to the trial end date, economic outcomes were inflated to more recent US dollar values. In addition, there have been no substantial changes in utilization, policy, or technology and, thus, the results of this study are relevant to inform contemporary practice, policy, and decision-making.

In conclusion, multidisciplinary care between podiatrists and physical therapists (uPOD plus physical therapist treatment) resulted in lower total costs and larger improvements in quality of life than uPOD. The directionality of the cost-effectiveness of uPOD plus physical therapist treatment was not sensitive to restricting cost valuation to PHP-specific costs or to those who completed treatment. The magnitude of cost-effectiveness was increased when individuals completed treatment as assigned, but was reduced when limiting the perspective to the health care sector and only PHP-specific costs.

## Supplementary Material

2025-0270_R1_PHP_RCT_Econ_Eval_PTJ_Supplementary_Material_FINAL_pzaf119(1)

## Data Availability

The datasets used and/or analyzed during the current study are available from the corresponding author on reasonable request and with appropriate institutional and ethics approval.

## CLINICAL TRIAL REGISTRATION

This trial was registered on ClinicalTrials.gov (Identifier: NCT01865734).
