# Supplementary material for: Cost-Effectiveness of Physical Therapist Treatment in Addition to Usual Podiatry Management of Plantar Heel Pain: Economic Evaluation of a Randomized Clinical Trial
Source: Phys Ther. 2025 Oct 3;105(11):pzaf119. doi: 10.1093/ptj/pzaf119 (PMC12581901; doi:10.1093/ptj/pzaf119)
Supplement: 2025-0270_R1_PHP_RCT_Econ_Eval_PTJ_Supplementary_Material_FINAL_pzaf119(1) [file 2025-0270_r1_php_rct_econ_eval_ptj_supplementary_material_final_pzaf119(1).pdf]

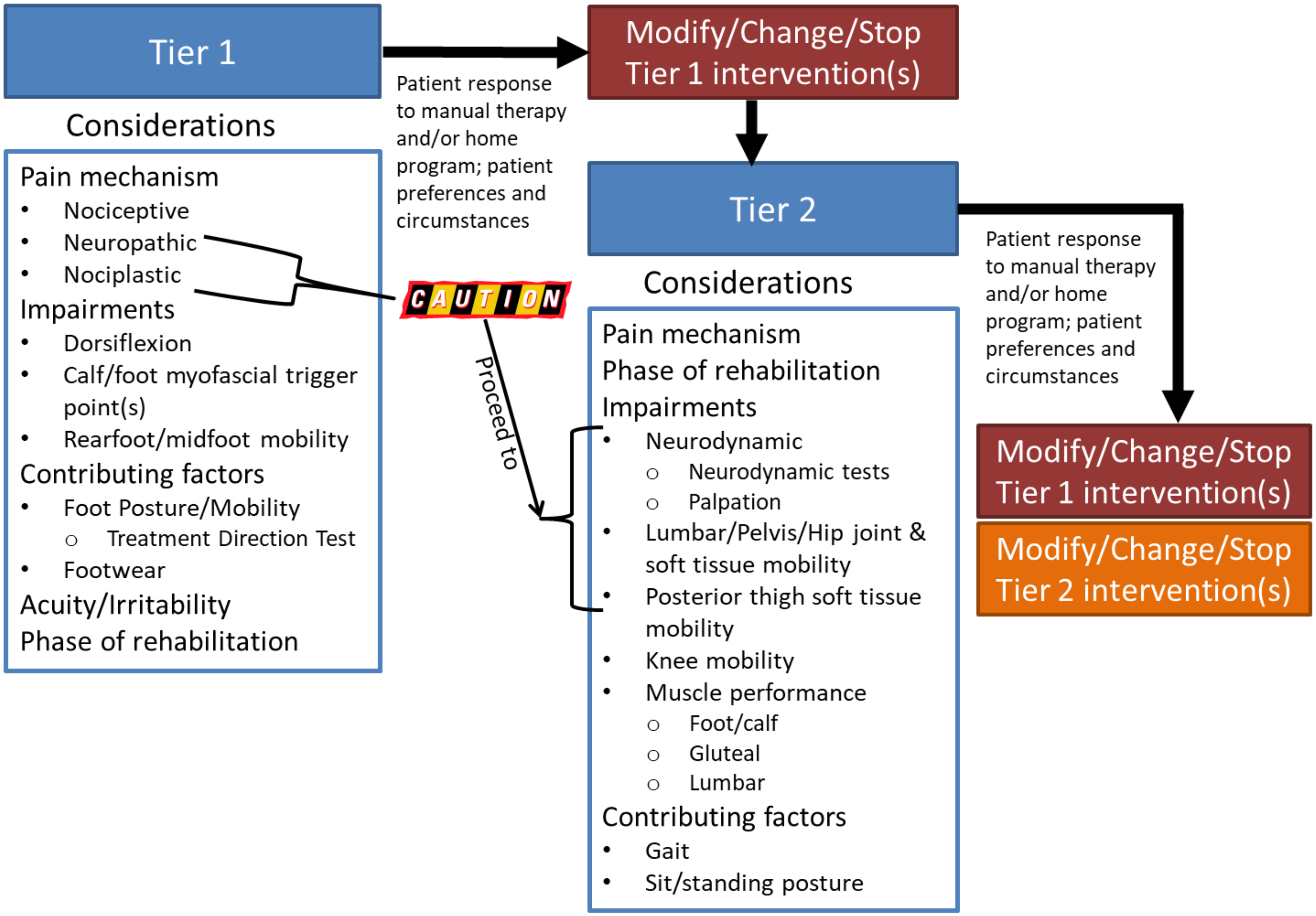

Supplementary Figure 1. Impairment-based and patient-centered decision-making guidance for the physical therapist intervention provided to the usual podiatric care plus physical therapist treatment (uPOD + physical therapy) group.

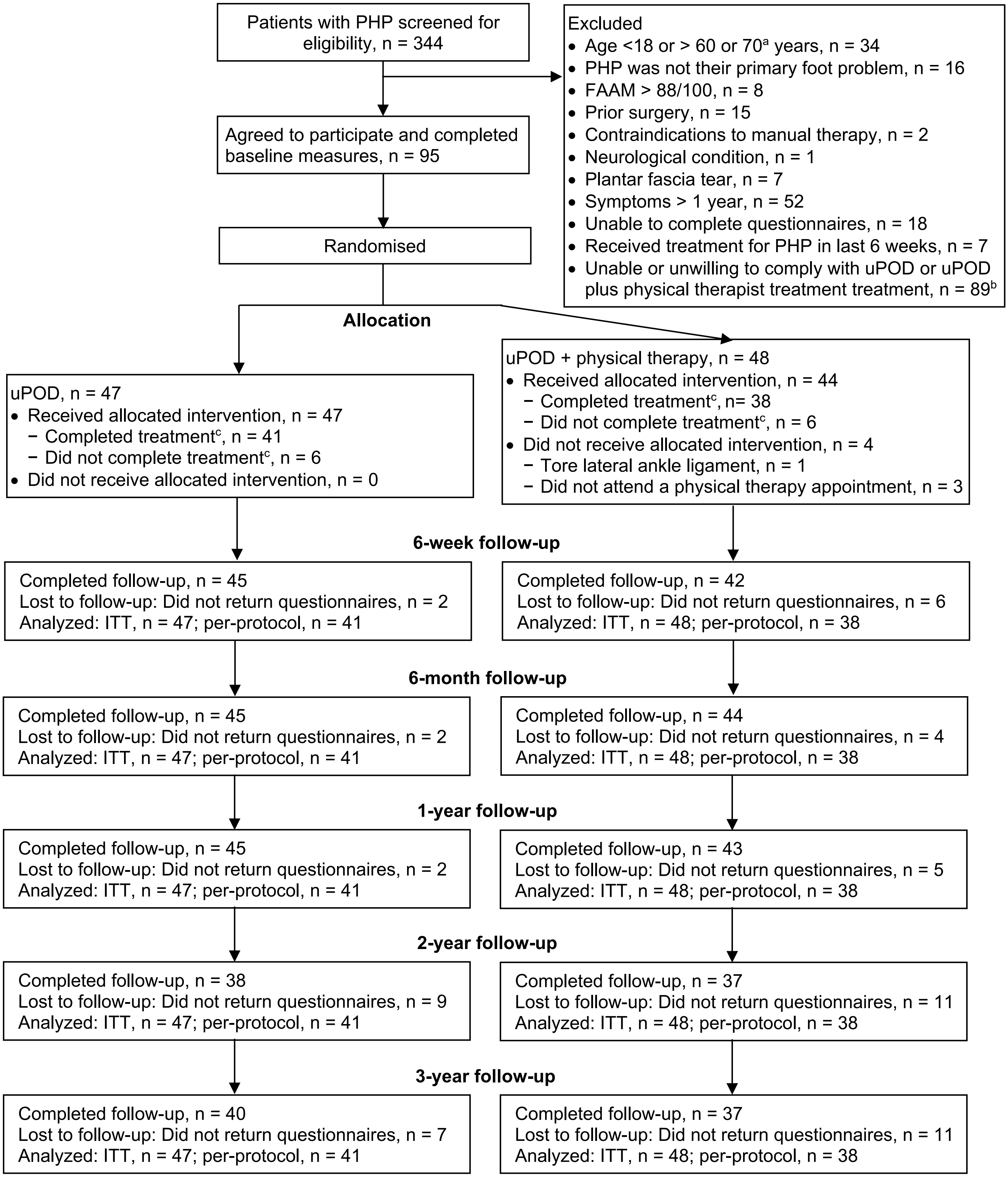

Supplementary Figure 2. Flow diagram of participant recruitment and retention. (a) Upper age limit was 60 years for the first 5 months of enrollment and increased to 70 for the next 32 months until enrollment ended; (b) Reasons provided were because the patient did not live near the clinic to attend regular appointments (n = 23), did not have time to participate (n = 27), was concerned about additional copayments or treatment-related costs (n = 19), or chose not to participate (n = 20); (c) Completed treatment were individuals who attended clinic appointments or follow-up according to the plan mutually set by the patient and provider as indicated in the participant's medical record or completed at least 4 visits with a physical therapist if assigned to the usual podiatric care plus physical therapist treatment (uPOD + physical therapy) group. Individuals that did not complete treatment were participants who failed to follow-up as planned. ITT **=** intention-to-treat, PHP **=** plantar heel pain, uPOD **=** usual podiatric care, FAAM **=** foot and ankle ability measure. Part of this figure was previously published in McClinton, SM, Heiderscheit, BC, McPoil, TG et al. Effectiveness of physical therapy treatment in addition to usual podiatry management of plantar heel pain: a randomized clinical trial. BMC Musculoskelet Disord. 2019;20(1):630. doi:10.1186/s12891-019-3009-y, which was published under a CC BY license. This figure was modified by adding the 2- and 3-year follow-up details.

Supplementary Table 1. Mean (SE, maximum) resource utilization related to PHP (first line of each category) and utilization not related to PHP where indicated.[a]

	**Baseline**				**Year 1**			
	Base Case		Per-Protocol		Base Case		Per-Protocol	
	**uPOD + physical therapy**	**uPOD**	**uPOD + physical therapy**	**uPOD**	**uPOD + physical therapy**	**uPOD**	**uPOD + physical therapy**	**uPOD**
**Healthcare Services (No. of visits)**								
Physical Therapist	0	0.02 (0.02, 1)	0	0.02 (0.02, 1)	4.1 (0.34, 11)	1.87 (0.59, 17)	4.68 (0.37, 11)	1.76 (0.61, 17)
Not PHP-related	0.42 (0.42, 20)	0.17 (0.16, 8)	0.53 (0.52, 20)	0.20 (0.19, 8)	0	0	0	0
Podiatry	0.23 (0.13, 6)	0.23 (0.09, 3)	0.26 (0.16, 6)	0.24 (0.10, 3)	2.17 (0.29, 12)	2.64 (0.32, 12)	1.76 (0.17, 5)	2.80 (0.36, 12)
Not PHP-related	0	0	0	0	0	0	0	0
Family Practice Physician	0.1 (0.06, 2)	0.02 (0.02, 1)	0.08 (0.06, 2)	0.02 (0.02, 1)	0	0	0	0
Not PHP-related	0.17 (0.07, 2)	0.19 (0.08, 3)	0.21 (0.08, 2)	0.22 (0.09, 3)	0.31 (0.14, 6)	0.13 (0.07, 3)	0.39 (0.18, 6)	0.15 (0.08, 3)
Acupuncture	0.02 (0.02, 1)	0	0.03 (0.03, 1)	0	0	0.06 (0.06, 3)	0	0.07 (0.07, 3)
Not PHP-related	0	0	0	0	0.17 (0.17. 8)	0	0.21 (0.21, 8)	0
Chiropractic	0.15 (0.13, 6)	0.15 (0.15, 7)	0.18 (0.16, 6)	0.17 (0.17, 7)	0.06 (0.05, 2)	0.34 (0.15, 5)	0.08 (0.06, 2)	0.39 (0.18, 5)
Not PHP-related	0.29 (0.14, 5)	0.43 (0.17, 6)	0.21 (0.12, 4)	0.34 (0.13, 3)	0.44 (0.22, 8)	0.49 (0.29, 12)	0.53 (0.26, 8)	0.27 (0.16, 6)
Osteopathic Manual Medicine	0.02 (0.02, 1)	0	0.03 (0.03, 1)	0	0.04 (0.04, 2)	0	0.05 (0.05, 2)	0
Not PHP-related	0.02 (0.02, 1)	0.02 (0.02, 1)	0.03 (0.03, 1)	0.02 (0.02, 1)	0.04 (0.04, 2)	0	0.05 (0.05, 2)	0
Massage	0.19 (0.12, 5)	0.11 (0.09, 4)	0.24 (0.15, 5)	0.12 (0.10, 4)	0.38 (0.17, 6)	0.4 (0.24, 9)	0.42 (0.21, 6)	0.39 (0.27, 9)
Not PHP-related	0.19 (0.10, 3)	0.09 (0.07, 3)	0.18 (0.11, 3)	0.07 (0.07, 3)	0.42 (0.21, 8)	0.15 (0.11, 4)	0.53 (0.26, 8)	0.17 (0.12, 4)
Orthopedic Physician	0.02 (0.02, 1)	0	0.03 (0.03, 1)	0	0	0.04 (0.04, 2)	0	0.05 (0.05, 2)
Not PHP-related	0	0	0	0	0	0.02 (0.02, 1)	0	0.02 (0.02, 1)
Reflexology	0.02 (0.02, 1)	0	0.03 (0.03, 1)	0	0	0.06 (0.06, 3)	0	0.07 (0.07, 3)
Not PHP-related	0	0	0	0	0	0	0	0
Surgery	0	0	0	0	0.02 (0.02, 1)	0.02 (0.02, 1)	0	0.02 (0.02, 1)
Not PHP-related	0.02 (0.02, 1)	0.02 (0.02, 1)	0.03 (0.03, 1)	0.02 (0.02, 1)	0.04 (0.03)	0.11 (0.04, 1)	0.03 (0.03, 1)	0.12 (0.05, 1)
**Home Services (No. of hours)**								
Housework/Yardwork help	2.04 (0.63, 20)	0.98 (0.28, 8)	2.42 (0.78, 20)	1.09 (0.31, 8)	0.67 (0.51, 22)	2.88 (1.61, 65)	0.84 (0.62, 22)	3.30 (1.84, 65)

Supplementary Table 1 (Continued). Mean (SE, maximum) resource utilization related to PHP and utilization not related to PHP where indicated.

	**Baseline**				**Year 1**			
	Base Case		Per-Protocol		Base Case		Per-Protocol	
	**uPOD + physical therapy**	**uPOD**	**uPOD + physical therapy**	**uPOD**	**uPOD + physical therapy**	**uPOD**	**uPOD + physical therapy**	**uPOD**
**Pharmaceuticals (No. of pills)**								
NSAID	35.73 (12.05, 400)	34.96 (9.54, 250)	29.61 (11.46, 360)	35.68 (10.25, 250)	59.83 (19.14, 708)	77.28 (18.37, 480)	67.42 (23.52, 708)	83.32 (20.91, 480)
Not PHP-related	29.06 (10.87, 365)	21.70 (7.16, 250)	36.71 (13.40, 365)	20.73 (7.99, 250)	106.27 (27.82, 912)	54.09 (16.51, 540)	127.66 (33.74, 912)	39.02 (13.94, 472)
Corticosteroid	0.88 (0.60, 21)	0.89 (0.61, 21)	0.55 (0.55, 21)	1.02 (0.70, 21)	4.17 (4.18, 200)	1.11 (0.64, 21)	0	1.27 (0.74, 21)
Not PHP-related	0.90 (0.65, 28)	14.47 (14.01, 680)	1.13 (0.80, 28)	16.59 (16.14, 680)	0.91 (0.61, 22.5)	1.36 (1.32, 64)	1.14 (0.79, 22.5)	1.56 (1.52, 64)
Analgesic	5.52 (3.18, 120)	5.74 (5.70, 270)	6.58 (3.99, 120)	6.59 (6.68, 270)	4.77 (4.15, 200)	2.68 (2.66, 126)	5.26 (5.08, 200)	3.07 (3.12, 126)
Not PHP-related	12.19 (6.37, 240)	15.40 (9.99, 450)	9.08 (5.08, 135)	17.66 (11.30, 450)	68.60 (28.45, 825)	58.62 (29.06, 1035)	67.24 (31.14, 825)	62.80 (32.99, 1035)
Glucosamine/ Chondroitin	7.5 (7.50, 360)	0	9.47 (9.02, 360)	0	0	0	0	0
Not PHP-related	0	0	0	0	0	0	0	0
Omeprazole/ Famotidine	0	0	0	0	0	0	0	0
Not PHP-related	7.79 (4.59, 180)	20.04 (6.52, 180)	7.47 (5.24, 180)	20.78 (7.22, 180)	18.08 (10.74, 444)	64.68 (20.18, 540)	19.37 (12.97, 444)	74.15 (22.38, 540)
**Productivity loss (No. of hours)**								
Absenteeism	4 (2.57, 120)	39.23 (37.56, 1800)	1.42 (0.77, 25)	44.39 (43.42, 1800)	3.79 (2.68, 118.75)	7.87 (6.99, 333.5)	3.36 (3.07, 118.75)	8.39 (7.93, 333.5)
Presenteeism	0.79 (0.48, 20)	0.56 (0.39, 17.5)	0.34 (0.27, 10)	0.55 (0.44, 17.5)	2.8 (1.92, 70)	2.06 (0.92, 36)	0.06 (0.06, 2.25)	2.27 (1.04, 36)

Supplementary Table 1 (continued). Mean (SE, maximum) resource utilization related to PHP and utilization not related to PHP where indicated.

	**Year 2**				**Year 3**			
	Base Case		Per-Protocol		Base Case		Per-Protocol	
	**uPOD + physical therapy**	**uPOD**	**uPOD + physical therapy**	**uPOD**	**uPOD + physical therapy**	**uPOD**	**uPOD + physical therapy**	**uPOD**
**Healthcare Services (No. of visits)**								
Physical **Therapist**	0.02 (0.02, 1)	0.13 (0.13, 6)	0.03 (0.03, 1)	0.15 (0.14, 6)	0.10 (0.10, 5)	0.43 (0.34, 15)	0.13 (0.13, 5)	0.49 (0.38, 15)
Not PHP-related	0	0.32 (0.32, 15)	0	0.37 (0.36, 15)	0.5 (0.51, 24)	0	0.63 (0.62, 24)	0
Podiatry	0.06 (0.04, 1)	0.02 (0.02, 1)	0.05 (0.04, 1)	0.02 (0.02, 1)	0.02 (0.02, 1)	0.04 (0.03, 1)	0.03 (0.03, 1)	0.05 (0.03, 1)
Not PHP-related	0	0	0	0	0	0	0	0
Family Practice Physician	0	0	0	0	0	0	0	0
Not PHP-related	0.35 (0.18, 6)	0.38 (0.16, 6)	0.39 (0.23, 6)	0.44 (0.18, 6)	0.06 (0.05, 2)	0.11 (0.06, 2)	0.08 (0.06, 2)	0.12 (0.07, 2)
Acupuncture	0.06 (0.06, 3)	0.09 (0.09, 4)	0.08 (0.08, 3)	0	0.06 (0.06, 3)	0	0.08 (0.08, 3)	0
Not PHP-related	0	0	0	0	0	0	0	0
Chiropractic	0	0	0	0	0	0.32 (0.32, 15)	0	0.37 (0.37, 15)
Not PHP-related	2.31 (1.28, 54)	1.12 (0.62, 26)	2.74 (1.60, 54)	0.65 (0.33, 10)	1.58 (1.16, 52)	0.21 (0.21, 10)	2 (1.40, 52)	0.24 (0.24, 10)
Osteopathic Manual Medicine	0	0	0	0	0	0	0	0
Not PHP-related	0	0	0	0	0	0	0	0
Massage	0	0.04 (0.04, 2)	0	0	0.04 (0.04, 2)	0.04 (0.04, 2)	0.05 (0.05, 2)	0
Not PHP-related	0.52 (0.29, 10)	0.26 (0.16, 6)	0.66 (0.36, 10)	0.29 (0.19, 6)	0.42 (0.30, 13)	0.02 (0.02, 1)	0.53 (0.36, 13)	0.02 (0.02, 1)
Orthopedic Physician	0	0.02 (0.02, 1)	0	0.02 (0.02, 1)	0	0	0	0
Not PHP-related	0.04 (0.04, 2)	0.06 (0.06, 3)	0.05 (0.05, 2)	0.07 (0.07, 3)	0	0.02 (0.02, 1)	0	0.02 (0.02, 1)
Reflexology	0.10 (0.10, 5)	0	0.13 (0.13, 5)	0	0	0	0	0
Not PHP-related	0	0	0	0	0	0	0	0
Surgery	0	0	0	0	0	0	0	0
Not PHP-related	0.02 (0.02, 1)	0.06 (0.04, 1)	0.03 (0.03, 1)	0.07 (0.04, 1)	0.06 (0.04, 1)	0.06 (0.04 ,1)	0.08 (0.04, 1)	0.07 (0.04, 1)
**Home Services (No. of hours)**								
Housework/Yardwork help	0.06 (0.06, 3)	0.14 (0.11, 5)	0.08 (0.08, 3)	0.15 (0.12, 5)	3.30 (3.32, 156)	1.21 (1.11, 52)	4.17 (3.96, 156)	1.39 (1.29, 52)

Supplementary Table 1 (continued). Mean (SE, maximum) resource utilization related to PHP and utilization not related to PHP where indicated.

	**Year 2**				**Year 3**			
	Base Case		Per-Protocol		Base Case		Per-Protocol	
	**uPOD + physical therapy**	**uPOD**	**uPOD + physical therapy**	**uPOD**	**uPOD + physical therapy**	**uPOD**	**uPOD + physical therapy**	**uPOD**
**Pharmaceuticals (No. of pills)**								
NSAID	0.25 (0.24, 12)	38.53 (12.67, 360)	0.32 (0.31, 12)	38.32 (14.22, 360)	0.38 (0.37, 18)	11.74 (5.71, 180)	0.47 (0.46, 18)	9.56 (5.64, 180)
Not PHP-related	39.65 (12.45, 365)	17.98 (8.71, 360)	43.5 (14.72, 365)	17.98 (10.20, 360)	33.13 (12.25, 365)	23.55 (7.96, 270)	41.84 (14.75, 365)	24.37 (9.39, 270)
Corticosteroid	0	0	0	0	0	0	0	0
Not PHP-related	1.41 (0.74, 28)	0.18 (0.12, 4.5)	1.36 (0.82, 28)	0.20 (0.14, 4.5)	0.98 (0.54, 16)	0.10 (0.09, 4.5)	0.82 (0.56, 16)	0.11 (0.11, 4.5)
Analgesic	0	0	0	0	0	0	0	0
Not PHP-related	9.81 (5.75, 225)	18.21 (12.52, 540)	12.39 (7.23, 225)	20.88 (14.59, 540)	14.94 (10.15, 450)	33.09 (19.49, 720)	18.87 (12.76, 450)	37.93 (22.44, 720)
Glucosamine/ Chondroitin	3.75 (3.75, 180)	0	4.74 (4.51, 180)	0	0	0	0	0
Not PHP-related	0	0	0	0	0	0	0	0
Omeprazole/ Famotidine	0	0	0	0	0	0	0	0
Not PHP-related	5.63 (4.17, 180)	26.81 (10.45, 360)	7.11 (5.21, 180)	30.73 (11.70, 360)	5.63 (4.17, 180)	25 (8.50, 270)	7.11 (5.21, 180)	28.66 (9.55, 270)
**Productivity loss (No. of hours)**								
Absenteeism	0	0.27 (0.26, 12.5)	0	0.30 (0.30, 12.5)	0	0	0	0
Presenteeism	0	0.04 (0.04, 2)	0	0	0	0.06 (0.06, 3)	0	0.07 (0.07, 3)

[a]NSAID = non-steroidal anti-inflammatory drug, PHP = plantar heel pain, QALY = quality-adjusted life-year, uPOD = usual podiatry care, uPOD + physical therapy = uPOD plus physical therapist treatment

Supplementary Table 2. Mean (SE) costs and health outcomes through 3 years in the base case, per-protocol, and PHP-specific costs analyses.[a]

	**Year 1**				**Year 2**				**Year 3**			
	Base Case		Per-Protocol		Base Case		Per-Protocol		Base Case		Per-Protocol	
**Cost and Health Outcomes**	**uPOD + physical therapy**	**uPOD**	**uPOD + physical therapy**	**uPOD**	**uPOD + physical therapy**	**uPOD**	**uPOD + physical therapy**	**uPOD**	**uPOD + physical therapy**	**uPOD**	**uPOD + physical therapy**	**uPOD**
**Cost outcomes in US $**												
Total Societal Costs	2913 (473)	3134 (497)	2664 (432)	3382 (551)	1036 (230)	1853 (568)	1103 (290)	2011 (653)	1216 (533)	1579 (495)	1532 (641)	1796 (553)
PHP-Related	1382 (234)	1573 (412)	1221 (152)	1683 (459)	102 (49)	223 (98)	127 (63)	208 (107)	123 (61)	214 (113)	156 (75)	234 (122)
Not PHP-related	1531 (431)	1560 (350)	1441 (405)	1699 (396)	932 (225)	1631 (561)	975 (288)	1804 (646)	1093 (512)	1365 (473)	1375 (622)	1562 (531)
Total Healthcare Sector Costs	2478 (458)	2397 (375)	2258 (412)	2580 (433)	951 (227)	1669 (561)	997 (289)	1832 (646)	1118 (517)	1431 (488)	1406 (627)	1637 (548)
PHP-related	947 (177)	836 (200)	817 (57)	881 (223)	19 (10)	39 (16)	21 (12)	29 (14)	25 (17)	66 (40)	31 (21)	76 (45)
Not PHP-related	1531 (431)	1560 (351)	1441 (403)	1699 (396)	934 (225)	1631 (561)	975 (288)	1804 (646)	1093 (512)	1365 (473)	1375 (622)	1562 (531)
Healthcare visit costs	1240 (299)	992 (233)	942 (157)	1068 (256)	395 (157)	640 (340)	482 (200)	704 (400)	697 (373)	482 (248)	880 (464)	553 (285)
PHP-related	672 (142)	597 (169)	570 (51)	624 (187)	6 (4)	22 (11)	6 (5)	21 (12)	9 (6)	50 (31)	11 (9)	56 (35)
Not PHP-related	568 (266)	396 (174)	371 (141)	444 (197)	388 (157)	617 (340)	475 (200)	685 (400)	687 (370)	433 (227)	869 (461)	495 (263)
Medication costs	778 (234)	1036 (278)	917 (294)	1114 (317)	367 (81)	477 (112)	289 (93)	520 (127)	174 (72)	607 (342)	217 (90)	692 (383)
PHP-related	7 (2)	15 (7)	7 (2)	17 (9)	0 (5)	2 (0)	6 (4)	0 (1)	1 (0)	1 (1)	0 (0)	0 (0)
Not PHP-related	770 (233)	1021 (273)	910 (292)	1097 (311)	367 (81)	470 (110)	289 (93)	514 (124)	174 (72)	606 (341)	217 (93)	691 (381)
Cost to patient, family, friends	815 (118)	793 (118)	769 (121)	848 (132)	274 (86)	728 (357)	332 (106)	778 (403)	345 (141)	488 (239)	436 (174)	549 (270)
Copayment/ coinsurance	462 (82)	368 (80)	398 (61)	398 (91)	189 (70)	554 (350)	227 (87)	606 (392)	245 (118)	342 (225)	310 (148)	392 (254)
PHP-related	269 (52)	224 (57)	238 (45)	241 (65)	12 (7)	11 (7)	15 (10)	1 (1)	16 (11)	15 (10)	20 (14)	17 (12)
Not PHP-related	193 (70)	144 (63)	161 (47)	158 (72)	178 (67)	543 (350)	212 (85)	605 (392)	230 (116)	327 (224)	290 (144)	375 (251)
**Transportation**[b]	26 (6)	10 (2)	25 (6)	11 (2)	2 (2)	1 (1)	4 (2)	1 (1)	0 (0)	0 (0)	0 (0)	0 (0)
Housework/Yardwork **help**[b]	146 (65)	204 (54)	167 (80)	232 (58)	44 (42)	96 (75)	55 (55)	102 (87)	76 (49)	86 (73)	96 (62)	98 (85)
Foot **orthoses**[b]	78 (16)	90 (20)	77 (17)	81 (19)	5 (2)	11 (6)	7 (4)	6 (4)	9 (4)	10 (6)	11 (5)	10 (7)
Devices to help symptoms/**function**[b]	105 (30)	119 (26)	102 (36)	124 (30)	32 (11)	67 (20)	41 (15)	61 (21)	14 (6)	50 (22)	17 (7)	46 (24)
Productivity **costs**[b]	80 (41)	312 (200)	34 (26)	352 (222)	0 (0)	9 (7)	0 (0)	9 (9)	0 (0)	2 (2)	0 (0)	2 (2)
**Health outcomes**												
EQ-5D-3L index	0.89 (0.02)	0.86 (0.02)	0.91 (0.02)	0.86 (0.02)	0.92 (0.02)	0.9 (0.02)	0.92 (0.02)	0.9 (0.02)	0.95 (0.01)	0.92 (0.01)	0.95 (0.02)	0.91 (0.02)
QALY	0.84 (0.02)	0.83 (0.02)	0.85 (0.02)	0.83 (0.02)	1.74 (0.03)	1.71 (0.03)	1.76 (0.03)	1.71 (0.03)	2.68 (0.04)	2.62 (0.04)	2.7 (0.05)	2.61 (0.04)
FAAM	0.9 (0.02)	0.87 (0.02)	0.94 (0.02)	0.88 (0.02)	0.93 (0.02)	0.94 (0.02)	0.93 (0.02)	0.95 (0.02)	0.97 (0.01)	0.95 (0.01)	0.96 (0.02)	0.95 (0.01)
NPRS	1.28 (0.24)	2.21 (0.35)	0.68 (0.15)	2.1 (0.35)	0.94 (0.21)	1.22 (0.26)	0.78 (0.23)	1.13 (0.28)	0.37 (0.13)	1.09 (0.27)	0.41 (0.17)	1.11 (0.29)

[a]**EQ-5D-3L = EuroQol 5-dimension 3-level, FAAM = foot and ankle ability measure activities of daily living subscale, = NPRS, numeric pain rating scale, PHP = plantar heel pain, QALY = quality-adjusted life-year, uPOD = usual podiatry care, uPOD + physical therapy = uPOD plus physical therapist treatment;** [b]Includes only costs related to PHP.

Supplementary Material: Plantar Heel Pain Cost and Consequences Questionnaire (PCQ)

ID____________________ Date:_____/_____/________

Treatment received at Des Moines University Clinic for your heel/bottom of foot pain

1. **In the past 3 months**, how much have you paid out of pocket (money not returned to you from your insurance) for your appointments at the Des Moines University Clinic? Please indicate either total cost or cost per visit for any that apply.

	Podiatry	Physical Therapy
**Co-payment**	$_________ ☐ Total cost, **OR** ☐ Cost per visit	$_________ ☐ Total cost, **OR** ☐ Cost per visit
**Co-insurance**	$_________ ☐ Total cost, **OR** ☐ Cost per visit	$_________ ☐ Total cost, **OR** ☐ Cost per visit
**Deductible**	$_________ ☐ Total cost, **OR** ☐ Cost per visit	$_________ ☐ Total cost, **OR** ☐ Cost per visit
☐ I had no insurance-related costs for my appointments.		

2. **In the past 3 months**, how much have you paid for travel to/from your appointments at the Des Moines University Clinic? Please indicate the miles travelled if you used a personal vehicle and/or the costs of public transportation.

**Personal vehicle**
_________**miles/visit**
**OR**
**Please calculate miles/visit for me using the following address(es)**<br>Address to appointment:
Address from appointment (write SAME if same address as to appointment):

**Public transportation**
$_________ ☐ Total cost, **OR** ☐ Cost per visit

Surgery

3. Have you had surgery since you started this study?

☐ Yes

☐ No

Surgery (please describe):	Date of surgery:

Medication, Supplements, and Vitamins

4. Obtain all of the pill/medication bottles and tubes that you have taken or have obtained for any reason ***in the past 3 months.*** Please list **ALL** of your medications, supplements, or vitamins using the information listed on the bottles/tubes or the best that you can remember. Please include both prescription and non-prescription medication and any prescriptions you have filled but have not used.

Medication, Supplement, or Vitamin	Strength	Number of pills, fluid oz., net wt. in bottle/tube	Number of bottles/tubes ***in past 3 months***	Mark if taken mostly for **your heel/ bottom of foot pain**
☐ None	--	--	--	--
Pill Example Name	750 mg	90 pills	2	☑
Liquid Example Name	1 mg/mL	4 fl oz	1	○
Cream Example Name	0.1%	60 g	3	○
				○
				○
				○
				○
				○
				○
				○
				○
				○
				○
				○
				○
				○
				○

5. ***In the past 3 months,*** how much have you paid for your medication or supplements that was **not** covered by your health insurance (out-of-pocket costs)?

$____________

Additional heel/bottom of foot pain treatment NOT provided at the Des Moines University Clinic

6. ***In the past 3 months***, please indicate **ANY** treatment you have received that was **NOT** provided at the Des Moines University Clinic.

	Number of visits	Mark the circle if this treatment was mostly for **your heel/bottom of foot pain**.
Physical therapy		○
Podiatry		○
Family practice physician		○
Orthopedic physician specialist		○
Osteopathic manual medicine		○
Chiropractic Care		○
Massage therapy		○
Steroid Injections		○
Acupuncture		○
Extracorporal shock wave therapy		○
Dry needling		○
Other:		○
☐ I have not received any treatment other than the treatment at Des Moines University Clinic		

Aids, devices, footwear, and footwear inserts

7. ***In the past 3 months,*** please indicate any purchases you have made to manage ***your heel/bottom of foot pain***.

Product	Cost ($)
Orthotics/arch support (circle one: CUSTOM or OVER THE COUNTER)	
Heel cup/cushion/pad	
Arch sleeve/sock	
Night splint or Strassburg sock	
Shoes or other footwear	
Topical ointment	
Massage tool	
Ice or heat bag/pad	
Other (please describe):	
☐ I have not purchased any items to help manage my heel/bottom of foot pain	

Impact on work

8. Are you in paid work?

<table>
<tr><th colspan="2">YES
Please answer questions a – e below</th><th>NO
Please answer additional question below</th></tr>
<tr><td>a. How many hours per week do you work?</td><td></td><td rowspan="3">Are you not working because of your heel/bottom of foot pain?
☐ Yes
☐ No</td></tr>
<tr><td>b. How much do you get paid?</td><td>$________/hour
OR
$________/year</td></tr>
<tr><td>c. In the past 3 months how much less have you worked because of your heel/bottom of foot pain?</td><td>________days
OR
________hours</td></tr>
<tr><td colspan="3">d. Occupation:</td></tr>
<tr><td colspan="3">e. Please describe any other ways you have restricted your work because of your heel/bottom of foot pain.</td></tr>
</table>

9. ***In the past 3 months***, how many **extra** hours would you have to work to catch up on tasks you were unable to complete in normal working hours due to your heel/bottom of foot pain? Enter "0" if you have not been less productive while at work because of your heel/bottom of foot pain.

_____________hours OR ☐ I have not been in paid work over the past 3 months

Impact on home duties

10. It may be that people with heel/bottom of foot pain who normally do household/yard tasks (cleaning the house, shopping, food preparation, taking care of children, raking, mowing, shoveling, etc.) must leave these tasks to be done by others due to their health problems.

In the past 3 months, have others taken over any of your household/yard tasks due to your heel/foot pain?

☐ Yes, family members or others (neighbor, volunteer, home health aide, etc) have taken over my household/tasks for an average of…	_________hours/**week**
☐ No, I have performed my usual household/yard tasks myself	

11. Have you had an injury or an issue not related to you heel/bottom of foot pain that affected any of your responses above?

☐ Yes, Please describe:

☐ No

THANK YOU FOR COMPLETING THIS QUESTIONNAIRE!
